# Biogenesis of Outer Membrane Vesicles Concentrates the Unsaturated Fatty Acid of Phosphatidylinositol in *Capnocytophaga ochracea*

**DOI:** 10.3389/fmicb.2021.682685

**Published:** 2021-05-21

**Authors:** Divya Naradasu, Waheed Miran, Shruti Sharma, Satoshi Takenawa, Takamitsu Soma, Nobuhiko Nomura, Masanori Toyofuku, Akihiro Okamoto

**Affiliations:** ^1^International Center for Materials Nanoarchitectonics, National Institute for Materials Science, Tsukuba, Japan; ^2^Department of Bioengineering, University of California, Los Angeles, Los Angeles, CA, United States; ^3^Graduate School of Life and Environmental Sciences, University of Tsukuba, Tsukuba, Japan; ^4^Microbiology Research Center for Sustainability, University of Tsukuba, Tsukuba, Japan; ^5^Graduate School of Chemical Sciences and Engineering, Hokkaido University, Sapporo, Japan

**Keywords:** *Porphyromonas gingivalis*, lipidomics, oral biofilm, blebbing, transmission electron microscopy, growth dependency, protein profile

## Abstract

Bacterial outer membrane vesicles (OMVs) are spherical lipid bilayer nanostructures released by bacteria that facilitate oral biofilm formation *via* cellular aggregation and intercellular communication. Recent studies have revealed that *Capnocytophaga ochracea* is one of the dominant members of oral biofilms; however, their potential for OMV production has yet to be investigated. This study demonstrated the biogenesis of OMVs in *C. ochracea* associated with the concentration of unsaturated fatty acids of phosphatidylinositol (PI) and characterized the size and protein profile of OMVs produced at growth phases. Transmission electron microscopy showed isolated spherical structures from cells stained with heavy metals, indicating the production of OMVs with a size ranging from 25 to 100 nm. Lipidome analysis revealed the presence of phosphatidic acid, phosphatidylethanolamine, phosphatidylcholine, and PI as the main lipids. Some unsaturated fatty acids of PI were present specifically in OMV and little in the outer membrane, suggesting that OMVs are generated from a specific region of the membrane through blebbing rather than a random process such as cell lysis. Furthermore, the lack of similar PI accumulation in the OMV of *Porphyromonas gingivalis* suggests that *C. ochracea* has a different biogenesis mechanism. The blebbing mechanism was further supported by higher OMV production occurring at the exponential phase in comparison to the stationary phase, where cell lysis is more likely to occur. Further, comparative protein profile of OMVs isolated under different growth phases may indicate that the OMV cargo does not largely vary with growth phases. The present study provides a basis for further understanding the roles of *C. ochracea* OMVs in oral biofilms as well as systemic diseases that *C. ochracea* involves.

## Introduction

Among the adapted modes for survival, the production of outer membrane vesicles (OMVs) by bacteria, particularly Gram-negative bacteria, plays a prominent role in interactions between themselves and the host (Jan, 2017; Lynch and Alegado, 2017). OMVs have gained recognition for their role in bacterial virulence. They are known to play similar or even more invasive roles as their parent cells in mediating adherence, host cell damage, modulation of host immune responses, and biofilm formation. Therefore, the biogenesis and function of bacterial OMVs in different human niches have drawn great interest (Toyofuku et al., 2019; Giordano et al., 2020).

The subgingival plaque is home to multispecies biofilms, and its development is strongly associated with the onset of chronic periodontitis (Kolenbrander et al., 2010). Although hundreds of bacterial species make up this structured biofilm (Saini et al., 2011), only a few of them are associated with disease progression (Paster et al., 2006). The increased concentrations of the gram-negative, anaerobic, and proteolytic bacteria such as *Porphyromonas gingivalis*, *Treponema denticola*, and *Tannerella forsythia* are strongly associated with symptoms of chronic periodontitis, and all three species secrete OMVs (Mohanty et al., 2019). OMVs, through their contributions to aggregation, communication, nutrient acquisition, and defense, play a key role in enhancing biofilm formation (Wang et al., 2015). The relationship between biofilms and OMVs by pathogens such as *Helicobacter pylori*, *Francisella*, *Pseudomonas aeruginosa*, *Vibrio cholerae*, and *Pseudomonas putida* from diverse niches have also been well validated (Yonezawa et al., 2011; van Hoek, 2013; Murphy et al., 2014).

A recent study revealed that *Capnocytophaga ochracea* is an important member of oral biofilms that occupies a wide band inside the oral biofilm periphery according to micron-scale organization of oral microbiomes obtained by metagenomic sequence analysis combined with spectral fluorescence imaging (Mark Welch et al., 2016). *Capnocytophaga* was identified as a genus with strong plaque specificity, which was 10-fold more abundant in plaque than at non-plaque sites. Moreover, it is also responsible for oral as well as systemic diseases in immunocompromised patients (Li et al., 2000; Mark Welch et al., 2016). Although the strong relevance with oral biofilm implies their OMV production, there is no study on OMV production in the genus *Capnocytophaga*. Given that OMV production occurs not only by biological mechanisms *via* blebbing, but also by cell lysis, multiple evidence such as the highest production in the exponential growth phase and different components than the outer membrane is required to confirm the biogenesis of OMV (Renelli et al., 2004; Toyofuku et al., 2019). In this study, we cultured *C. ochracea* and examined the production of OMVs at different growth phases using transmission electron microscopy (TEM), single particle tracking analysis, lipidomics, and polyacrylamide gel electrophoresis. Moreover, *P. gingivalis* was also cultured to obtain purified OMVs and analyze its lipid components.

## Materials and Methods

### Bacterial Growth Media and Culture Conditions

*Capnocytophaga ochracea* ATCC 27872 was grown in 80 mL of DSMZ 340 medium supplemented with 1 g/L sodium bicarbonate at 37°C. The medium, excluding glucose and hemin, was autoclaved for 15 min at 121°C before the culture. Glucose and hemin were separately prepared and filter-sterilized prior to inoculation. To maintain anaerobic growth conditions, 20 min of N_2_/CO_2_ (80:20 v/v) gas sparging was performed prior to culture inoculation. Sterility during sparging was maintained by using 0.22 μm sterile syringe filters. The medium was inoculated with *C. ochracea*, incubated at 37°C, and allowed to achieve one of the five stages of growth explored in this study. The initial pH of the medium was 6.9 ± 0.1. Growth phases were determined by the pH of the culture using a *C. ochracea* growth curve relating pH and optical density (OD_600_) with time. The growth phases explored in this study were the lag phase (pH 6.5), early exponential phase (pH 6.0), mid-exponential phase (pH 5.7), late exponential phase (pH 5.3), and stationary phase (pH 5.1). *P. gingivalis* (strain ATCC BAA-308/W83) was also grown anaerobically, similar to *C. ochracea* in Gifu anaerobic medium. The culture was grown at 37°C until the growth reached the late exponential phase, in which the OD_600_ was 1.0. We used *P. gingivalis* W83 to compare with *C. ochracea* because strain W83 is a pathogenic oral bacterium, contributor to periodontal disease, and produces OMVs (Gui et al., 2016).

### Transmission Electron Microscopy

*Capnocytophaga ochracea* cells were collected from 2–4 mL of pre-cultures in the exponential growth phase by centrifugation at 6,000 × *g* for 10 min and immediately fixed in solutions containing 2% paraformaldehyde and 2.5% glutaraldehyde on ice. After fixation, all manipulations were conducted in 2 mL Eppendorf tubes. Washing was completed with 5 × 1.5 mL washes with gentle resuspension and centrifugation (6,000 × *g*, 5 min) in 50 mM Na^+^-HEPES (pH 7.4, 35 g/L NaCl). Sequential ethanol gradient dehydration and resin embedding procedures were conducted according to a previously described method (McGlynn et al., 2015). The obtained resin blocks were sectioned at 80 nm with a diamond knife (DiATOME, ultra 35°), and floating sections were mounted on copper microgrids (Nishan EM). Thin sections were examined and imaged using a JEM-1400 microscope operated at an acceleration voltage of 80 kV. To visualize the membrane structures, the samples were stained with 2% uranyl acetate at room temperature (25°C) for 15 min, followed by secondary staining with Lead stain solution (Sigma-Aldrich Co., Tokyo, Japan) at room temperature for 3 min, and then washed with distilled water. Finally, samples were post-fixed with 2.0% osmium tetroxide.

### Isolation and Purification of Outer Membrane Vesicles

When the desired growth phase for a given condition was achieved, the culture medium was centrifuged for 10 min at 7,800 rpm and 4°C. The resulting supernatant containing outer membrane vesicles was passed through 0.22 μm filters to remove any cell debris. The filtered supernatant was ultracentrifuged for 2 h at 126,000 × *g* at 4°C. Following ultracentrifugation, the supernatant was decanted, and the pelleted outer membrane vesicles (OMVs) were re-suspended and stored in phosphate buffered saline (PBS) at 4°C until further purification. OMV purification was conducted using an iodixanol (OptiPrep) density gradient. The 60% iodixanol stock solution was diluted to 35, 30, 25, 20, 15, and 10% in PBS. All the stored OMVs for a given condition were combined and ultracentrifuged for 2 h at 140,000 × *g* at 4°C. This collective pellet was resuspended in 35% iodixanol solution. The six iodixanol density layers were carefully pipetted in decreasing order of percent density into a 7 mL ultracentrifuge tube with the sample containing 35% solution at the bottom and 10% solution at the top. The layers were loaded from the top, with each layer consisting of 1 mL of the desired weight solution. Tubes containing density layers were ultracentrifuged for 16 h at 140,000 × *g* and 4°C using a swing rotor. Following ultracentrifugation, six 1 mL fractions were taken from each tube and placed into six different Eppendorf tubes to separate the layers. Isolated MVs were placed on Thin Carbon film TEM grids (ALLIANCE Biosystems, Osaka, Japan) or formvar-coated copper grids (200 mesh) for TEM observations. Further, TEM observations of isolated OMVs were carried out by staining with 0.5 % sodium molybdate for 60 sec using JEOL-1010 transmission electron microscope. For Nanosight, samples were stained with EM stainer (Nisshin EM, Tokyo, Japan), rinsed and observed using a Hitachi H- 7650 transmission electron microscope (Hitachi, Tokyo, Japan).

### Quantification of Outer Membrane Vesicles

Total protein concentrations were measured for all fractions using a bicinchoninic acid (BCA) assay kit according to the manufacturer’s instructions to determine the fractions containing the greatest concentration of OMVs. The OMV isolation was conducted from the culture stages where the pH of the growth medium was pH 6.5, 6.0, 5.7, 5.3, and 5.1. No significant OMVs were observed in the cultures collected at pH 6.5 and pH: 6.0 (data not shown). The purified OMV fractions collected at different growth phases were subjected to measurement of the concentration and particle size using a NanoSight NS300 (NanoSight Ltd., Amesbury, United Kingdom).

### DNase Treatment and DNA Quantification

To a 100 μL of OMV sample, 2 μL of DNase [13 units (U)/μL] was added to a final concentration of 2 U to treat the external DNA of OMV. This sample was stored at 37°C for 30 min, followed by DNase deactivation by heating the sample at 80°C for 10 min. Later, OMV DNA was extracted from DNase treated and untreated OMVs using the Isoplant-II DNA extraction kit according to the manufacturer’s instructions. The extracted DNA was quantified by using a NanoDrop UV-Vis spectrophotometer (Thermo Scientific NanoDrop 2000, Japan).

### Lipidomics and Proteomic Analysis

This study compared the lipid profiles of Inner membrane (IM), Outer membrane (OM), and OMVs. For IM and OM, the cell membrane was extracted by physical disruption using a bead shocker (MB3000, Yasui Kikai, Japan). Initially, cell pellets were collected from a 500 mL culture and stored at −80°C until further use. The pellet was then resuspended and washed in 20 mM Tris–HCL pH-8 buffer by centrifugation at 7,800 rpm for 10 min at 4°C, and the pellet weight was measured. Sterilized zirconium beads (0.1 mm) were added to the pellet in a 1: 2 (w/w) cells to beads ratio and resuspended in 15 mL. The cell and bead mixture was transferred to the bead shocker tube and cells were broken for 20 min at full speed (2,700 rpm, 30 s on time, 30 s OFF time, seven cycles). Beaded cells were placed on ice to allow the beads to settle down at the bottom, and centrifuged at 7,800 rpm for 15 min at 4°C to remove the cell debris. The bead-free supernatant was further centrifuged at low speed (2,000 rpm, 10 min at 4°C) to remove cell debris and repeated twice. Cell-free extract (supernatant) was transferred to an ultracentrifuge tube (15 mL in each tube). Cell envelopes were obtained from the supernatant after centrifugation at 12,000 × *g* for 1 h at 4°C in a Hitachi-type S50A-2130 rotor. The cells were washed with Tris-buffer and the cell envelope fraction (1 mL suspension) was loaded onto a discontinuous sucrose gradient [1 mL each of 55, 50, 40, and 30% (w/w) sucrose] and centrifuged in a Hitachi-type S50ST-2069 rotor for 12 h at 16,000 g. Cell walls were isolated from middle fractions. OM and IM membrane fractions were confirmed by SDS–polyacrylamide gel electrophoresis, with proteins stained by Gel Code Blue Safe Protein Stain (Thermo Fisher Scientific).

For lipidome analysis, the bacterial OMV solution was washed with water and transferred to a test tube. Two milliliters of methanol and 2 mL of chloroform were added, and the mixture was shaken for 30 s. The mixture was centrifuged at 3,000 rpm for 5 min, and then the lower layer (organic layer) was transferred to a new tube. Further, 2 mL of chloroform was added to the residue in the upper layer, shaken for 30 s, centrifuged at 3,000 rpm for 5 min, and then transferred to the lower layer in the first test tube in duplicate. The organic layer was dehydrated under a stream of nitrogen. The residue was reconstituted with dichloromethane/methanol (0.5 mL of, 4/1, v/v) to prepare analytical samples. Chromatographic separation of phospholipids was achieved on an Inertsil SIL-100A column (250 mm length × 2.1 mm id, 3 μm, GL Sciences Inc., Tokyo, Japan). Eluent A was a 90% methanol solution containing 0.1% ammonia, and eluent B was dichloromethane. Separation was performed by gradient elution at a flow rate of 0.5 mL/min with a Nexera UHPLC system (Shimadzu Corporation, Tokyo, Japan) and eluted with linear gradients from 93% eluent B (0 to 3.5 min), 93 to 74% B (3.5 to 9.0 min), 74 to 25% B (9.0 to 11.0 min), 25% B (held for 5 min), and 93% B (0.1 min, held for 6 min). Detection was conducted on a Triple Quad5500 mass spectrometer (SCIEX, Framingham, United States) with electrospray ionization. A Positive ion–mode precursor ion scan was used for PC with a target ion of m/z 184. A Positive ion–mode neutral loss scan was used for PE, PS, and PG with target ions of m/z 141, 185, and 172, respectively. Negative ion–mode precursor ion scans were used for PA and PI with target ions of m/z 153 and 241, respectively. Quantitation plots of each phospholipid peak area versus the concentration of phospholipids in standard solution were constructed using Analyst 1.7. and least squares linear regression was applied to the data. Concentrations of phospholipids in the analysis samples were calculated using the quantitation plots. The composition ratio of the sum of the alkyl chain lengths and the sum of the degrees of unsaturation of the fatty acids was calculated. OMV proteins at different growth phases were checked using SDS–polyacrylamide gel electrophoresis, with proteins stained by colloidal Coomassie blue (Thermo Fisher Scientific) (Neuhoff et al., 1988).

## Results

### OMVs Production by *Capnocytophaga ochracea*

**FIGURE 1:**
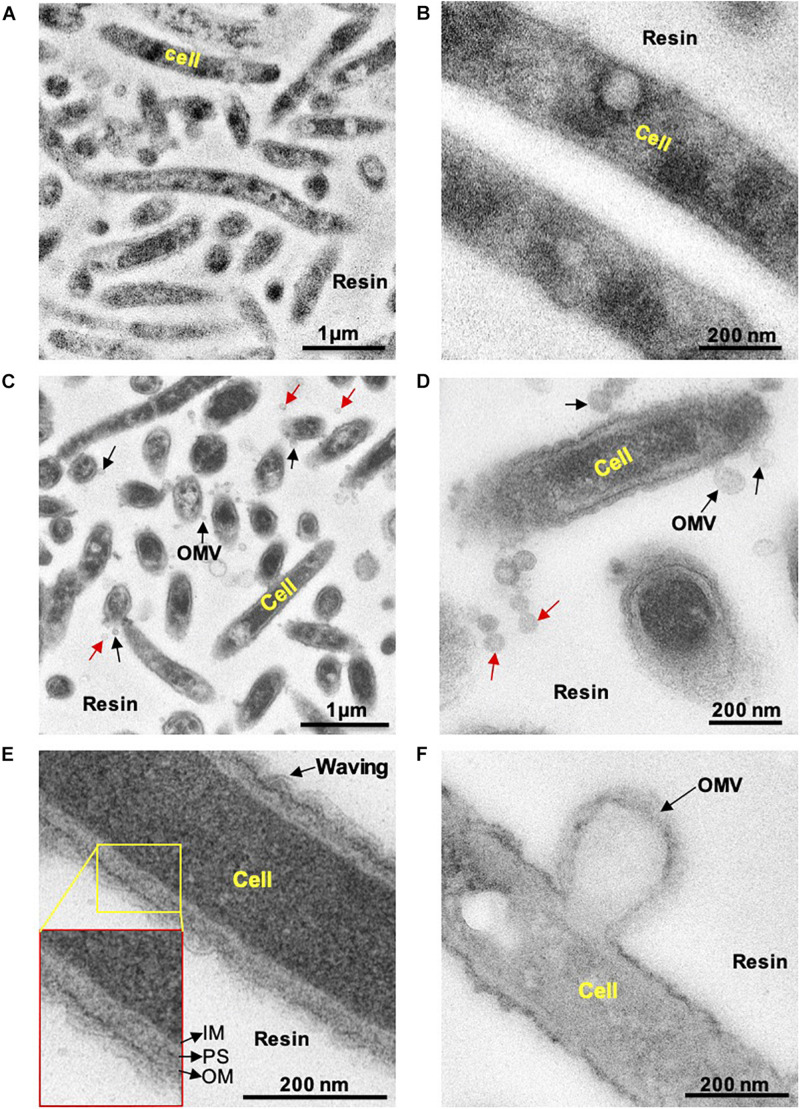
Transmission electron microscopy observation of thin sections of *C. Ochracea* cells. The TEM images of the specimens that were untreated and treated with Pb, U, and Os heavy metals. **(A)** General view of cells without heavy metal staining. **(B)** Magnified view of the cell surface without heavy metal staining **(C)** A general view of cells with heavy metal staining showing OMVs. **(D)** Magnified view of the cell surface with heavy metal staining showing OMVs. Black and red arrows indicate cell-attached and non-attached OMVs, respectively. **(E)** Magnified image of the cell and cell envelope showing membrane waving behavior. The inset shows the inner membrane, periplasmic space, and outer membrane. IM, inner membrane; PS, periplasmic space; OM, outer membrane. **(F)** Magnified image of OMV-forming cells.

First, we examined whether *C. ochracea* cells could produce OMVs through microscopy. Late exponential growth phase cells were used for the observation of OMVs, where thin sections of cells were subjected to TEM analysis. Prior to TEM observations, the cells were treated with Pb, uranium, and osmium heavy metals. The combination of heavy metals produced strong staining of the lipid bilayer and outer vesicle surfaces, and samples with heavy metal staining showed strong contrast in the cell membrane in comparison to cells without staining (Figure 1). The results revealed that stained particles had diameters mostly ranging between 25 and 100 nm, and their membranes showed the same structure and staining profile as the outer cell membrane (Figure 1), indicating the release of OMVs in *C. ochracea*. Although many OMVs were observed out of the cell (indicated by red arrows, Figures 1C,D), in a few cases, it was possible to visualize the vesicles attached to the cell surface (indicated by black arrows, Figures 1C,D) or precisely at the moment of formation and before they were detached from the cell (Figures 1C,D,F). It is difficult to confirm whether OMVs attached to the cell surface were secreted either by the same cell or from the other cells. However, the waving behavior of the outer membrane and OMVs secretion from the cell surface after outer curvature formation suggests that blebbing was the mode of OMV formation in *C. ochracea* (Figures 1E,F). In addition, no dividing cells were observed, and intact cells appeared to secrete OMVs. In TEM sections, it was observed that several of the vesicles showed an elongated shape at the time of formation and expulsion from the cell; however, vesicles became more round once liberated (Figures 1C,D). Based on these observations, it can be proposed that vesicles were derived from the outer membrane of *C. ochracea* and less likely to result from explosive cell lysis (Turnbull et al., 2016), a mode of membrane vesicle formation where the peptidoglycan cell wall degrades mostly during the stationary growth phase or forced vesiculation.

### Lipidome Comparison of Purified OMVs and Cell Membranes in *Capnocytophaga ochracea* and *Porphyromonas gingivalis*

**FIGURE 2:**
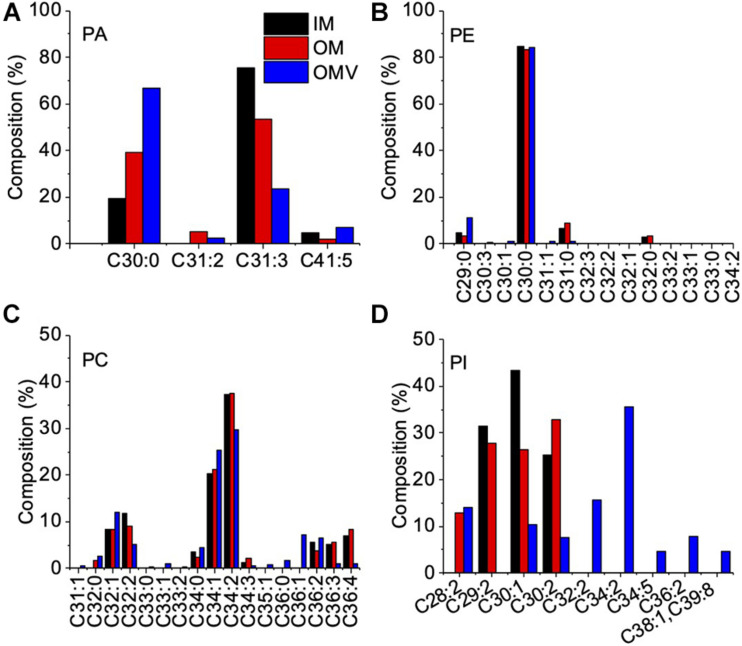
Fatty Acid Composition of Phospholipids in the IM, OM, and OMVs of *C. ochracea.* Percentage composition of fatty acids in phospholipids of **(A)** PA, **(B)** PE, **(C)** PC, and **(D)** PI. Fatty acids were identified by comparing their retention times with standards. Nonacosanoic acid (C29:0), Triacontanoic acid (C30:0), Hentriacontanoic acid (C31:0), Dotriacontanoic acid (C32:0), Tritriacontanoic acid (C33:0), Tetratriacontanoic acid (C34:0), Hexatriacontanoic acid (C36:0).

To examine the OMVs biogenesis mechanism, we compared the outer membrane and OMV lipid components using purified OMVs. First, OMVs were isolated from the culture supernatant (Supplementary Figure 1A). The pellet obtained after ultracentrifugation was resuspended in buffer, and a small aliquot was negatively stained and examined by TEM. To remove a small amount of bacterial debris with OMVs (Supplementary Figure 1B), we purified OMVs using an OptiPrep density gradient (Supplementary Figure 1C), and the cell debris was removed (Supplementary Figure 1D). A comparison of the IM and OM lipid composition of *C. ochracea* and its OMVs by lipidomics was performed, which showed the presence of commonly found bacterial lipids such as phosphatidic acids (PA), phosphatidylethanolamine (PE), phosphatidylcholine (PC), phosphatidylglycerol (PG), and phosphatidylinositol (PI), where PE was the most abundant lipid detected in all samples with a composition of more than 99% (Table 1). Further analysis of fatty acids showed that the fatty acids of PG were not detected by chromatography, whereas less significant changes in fatty acids of PA, PE, and PC were observed in IM, OM, and OMVs (Figures 2A–C). However, a significant difference was observed in the case of PI, where more unsaturated fatty acids were highly enriched in OMVs lipids compared to the IM and OM of *C. ochracea* (Figure 2D). The PI with unsaturated fatty acids specifically concentrated in OMVs suggests that the enrichment of PI leads to the blebbing of the outer membrane in *C. ochracea.* Furthermore, given that the biophysical characteristics of membrane lipids dictate membrane curvature and fluidity and thus probably play a key role in OMV biogenesis, it is likely that increased membrane flexibility from areas of enrichment of these fatty acids may play a role in promoting OMV biogenesis (Schwechheimer and Kuehn, 2015; Bogdanov et al., 2020).

**TABLE 1:** Phospholipid contents of purified IM, OM, and OMVs (ng/Sample).

	*C. ochracea*			*P. gingivalis*		
		
Phospholipid	IM	OM	OMVs	IM	OM	OMVs
Phosphatidic acids (PA)	87.0	76.8	96.0	1,686	1,394	148.8
Phosphatidylinositol (PI)	284	326	202	396,000	208,000	18,720
Phosphatidylcholine (PC)	13.1	10.54	108	9.36	7.38	3.54
Phosphatidylglycerol (PG)	348	474	10.8	6,780	2,620	426
Phosphatidylethanolamine (PE)	78,800	89,600	49,400	3,620	1,096	1,118

In this study, we also conducted the same lipidomics analysis for *P. gingivalis*, a well-known OMV-producing oral pathogen, to compare *C. ochracea.* The lipidomics of *P. gingivalis* revealed differences between bacterial lipid contents, such as PA, PE, PC, PG, and PI in IM, OM, and OMVs (Table 1). In addition, significant differences in fatty acid levels were observed (Supplementary Figure 2), and there was heterogeneity of the lipidome in *P. gingivalis* as observed in *C. ochracea*. However, even when PI lipid contents were highest compared to other phospholipids in all samples of *P. gingivalis*, the difference in unsaturated fatty acids was not significant unlike *in C. ochracea* (Supplementary Figure 2E), suggesting that different lipids from PI may be involved in the biogenesis of OMVs in *P. gingivalis.* Earlier anionic lipopolysaccharide (A−LPS) through deacylation of A−LPS has been proposed to increase the curvature leading to OMV formation in *P. gingivalis* (Gui et al., 2016). In this study, we observed the difference in PA contents of OMVs in *P. gingivalis.* Further investigation are needed to explore their possible role in OMV biogenesis, as PA is also known to influence membrane curvature (Zhukovsky et al., 2019). Additionally, given OMVs from *P. gingivalis* has DNA inside (Bitto et al., 2017), we also checked the DNA concentration in OMVs from *C. ochracea.* DNA was detected not only in the DNase treated (232 ng/μL) but also untreated (236 ng/μL) OMVs, suggesting the encapsulation of cellular DNA while biogenesis of OMVs in both strains.

### OMV Size and Protein Profile at Different Growth Phases

**FIGURE 3:**
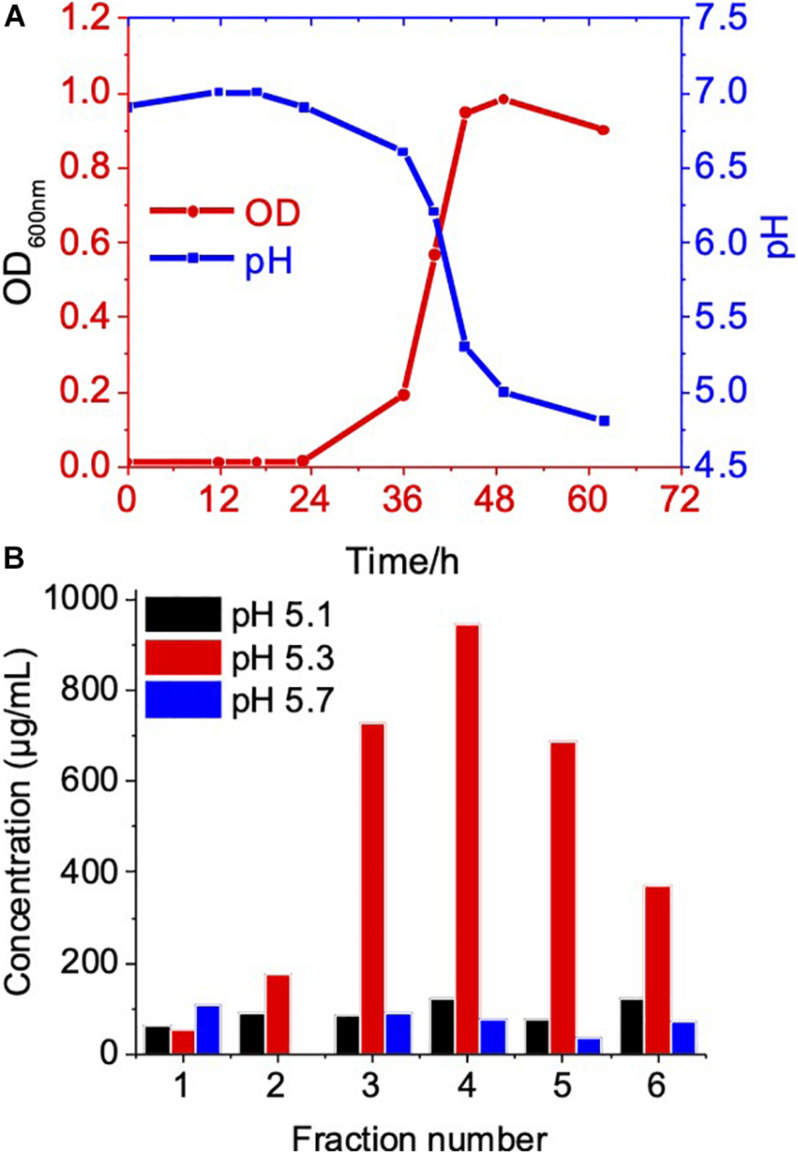
Growth phase related OMV production in *C. ochracea.* **(A)** Time vs Growth curve of *C. ochracea* and pH profile of the nutrient medium measured during the growth. **(B)** Total protein concentration of OMV from gradient purified fractions of stationary phase (pH: 5.1), late exponential phase (pH: 5.3), and mid-exponential phase (pH: 5.7).

The biogenesis of OMV likely occurs during the exponential growth phase (Tashiro et al., 2010; Klimentova and Stulik, 2015); as a result, a high number of budding OMVs on the cell surfaces during the log phase suggested the biogenesis of OMVs (Figures 1C,D). Meanwhile, during the stationary phase of bacterial growth, the yield and composition of OMVs could be altered (Tashiro et al., 2010; Klimentova and Stulik, 2015). Therefore, we further compared OMV production and protein components of *C. ochracea* at different growth phases. Growth phases were determined by the pH of the culture, as the *C. ochracea* growth curve showed a relationship between pH and optical density (OD_600_) with time, as shown in Figure 3A. The growth phases explored in this study were the lag phase (pH 6.5), early exponential phase (pH 6.0), mid-exponential phase (pH 5.7), late exponential phase (pH 5.3), and stationary phase (pH 5.1). OMVs were isolated after a specific pH and OD_600_ was reached and isolated as previously described. The quantity of OMVs was characterized by measuring the total protein concentration from the gradient purified fractions (Figure 3B). No visible pellet of OMV production was observed after ultracentrifugation (data not shown) from the cell culture taken in the lag phase (pH 6.5) and early exponential phase (pH 6.0). However, the OMV yield measured in terms of protein concentration started to increase initially from mid-exponential (pH: 5.7) and higher concentrations of OMVs at late exponential (pH: 5.3); however, it started to decrease at the stationary phase (pH: 5.1), as shown in Figure 3B. The cells in the stationary phase displayed lower levels of vesiculation, which suggests that active growth promotes OMV production in *C. ochracea*. Furthermore, the OMVs were quantified using Nanosight to measure the number of particles yielded at different growth phases of *C. ochracea.* The collected fractions of purified OMVs were tested for qualitative assessment using the lipid layer dye FM4-64 fluorescence (Supplementary Table 1). Fluorescence was measured after incubation with the lipid probe FM4-64, and fractions that showed higher fluorescence were subjected to quantitative analysis of the OMV number of particles by Nanosight.

**FIGURE 4:**
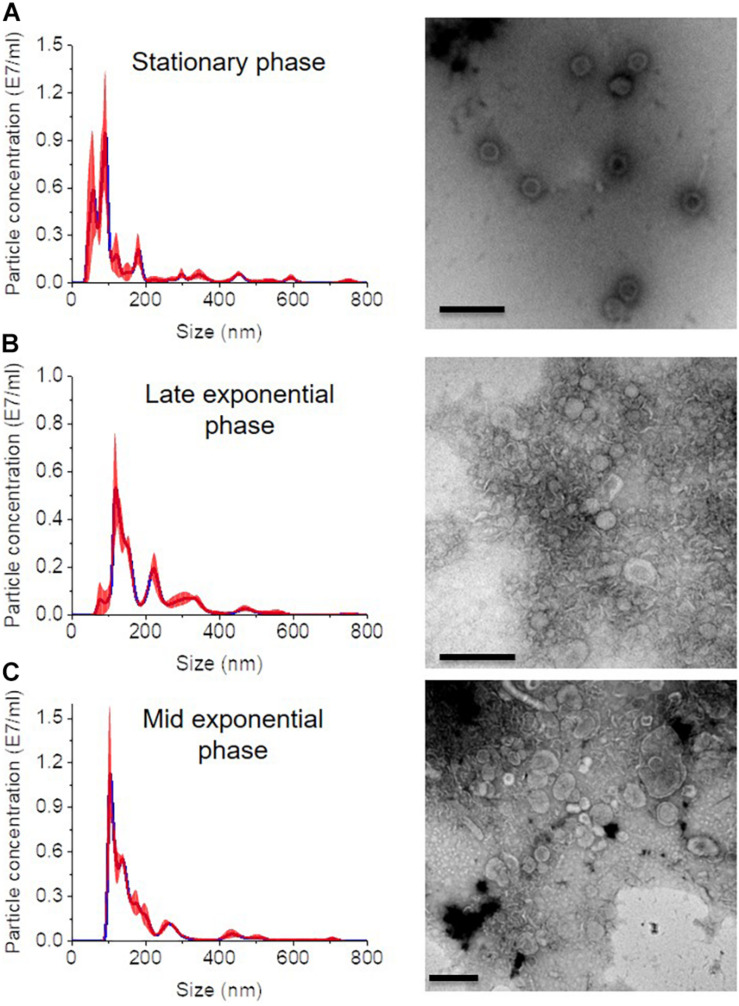
Nanosight analysis and respective TEM images of OMVs at different growth phases **(A)** stationary phase (pH: 5.1) **(B)** late exponential (pH: 5.3) and **(C)** mid-exponential phase (pH: 5.7). Each sample is measured three times. Scale bar, 200 nm.

**FIGURE 5:**
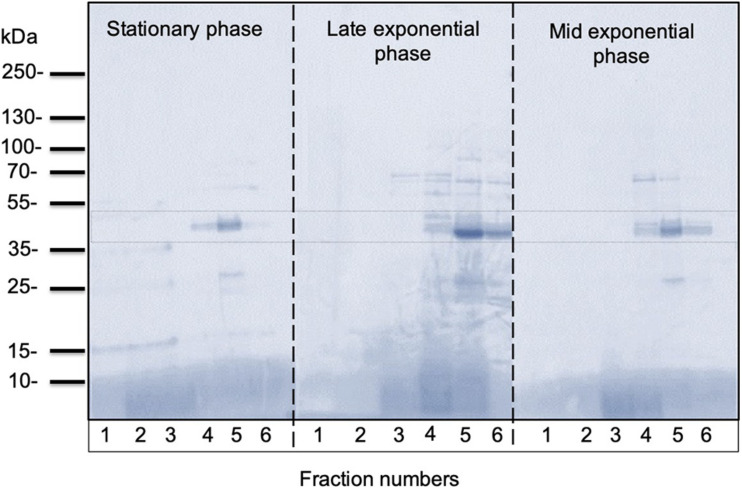
Representative SDS-PAGE analysis of OMVs isolated from *C. ochracea.* Protein profiles of OMVs isolated from cultures of different growth phases; stationary phase (pH: 5.1), late exponential phase (pH: 5.3), and mid-exponential phase (pH: 5.7). The gel was stained with Coomassie Brilliant Blue.

In all growth phase cases, OMV fractions ranging from to 3–5 showed higher fluorescence. Nanosight analysis revealed that OMVs of the stationary phase (pH: 5.1) contained OMVs ranging mostly between 20 and 150 nm in size. OMVs purified from the late exponential (pH: 5.3) and mid-exponential phase (pH: 5.1) showed OMVs size starting around 50 nm and contained aggregations of OMVs, thus showing a slightly larger diameter (Figure 4) in particle analysis and TEM images. Furthermore, multiple sized populations were contained in all three growth phases, indicating that there is heterogeneity in the size of OMVs pertaining to the growth phase (Figure 4). Thereafter, the purified OMV fractions obtained from the different growth phases was analyzed using sodium dodecyl sulfate-polyacrylamide gel electrophoresis (SDS-PAGE) to compare the enriched proteins in OMVs. Fractions 4–6 of all growth phases showed significant protein expression in SDS-PAGE with a molecular weight ranging between and 35–55 kDa (Figure 5). Other low-intensity bands were present in fractions 4 and 5 at pH 5.3, whereas the intensity was much lower at pH 5.1 and pH 5.7 conditions. Overall, results from the protein profile assay indicate that the OMV cargo does not vary significantly with different growth phases.

## Discussion

Several gram-negative bacteria from oral pathogens are known to produce OMVs. Hence, this study is the first to propose the mechanism of OMV biogenesis in periodontitis causing *C. ochracea*. The results of this study showed that the unsaturated and branched-chain fatty acids of PI lipids in the *C. ochracea* OMVs were enriched, indicating increased membrane fluidity and likely responsible for biogenesis of OMVs. The heterogeneity of phospholipids in bacterial membranes has already been confirmed (Matsumoto et al., 2006) and differential lipid compositions identified in different bacterial species support the hypothesis that OMVs are formed at specific OM regions as a result of compartmentalization or remodeling of the OM (Haurat et al., 2015). Hence, in such a model of OMV biogenesis, specific phospholipids accumulate in the outer leaflet of the OM, which then results in an asymmetric expansion of the outer leaflet and consequently promotes outward bulging of the OM, which finally pinches off to form an OMV. Moreover, it has been reported earlier that the composition of acyl chains or head groups can alter bilayer fluidity and stability, and this alteration is considered important in response to environmental perturbation (Sonnino and Prinetti, 2010). However, the enrichment of unsaturated and branched-chain fatty acids in the OMVs in this study and a few earlier studies suggest that lipids that increase membrane fluidity are shed *via* OMV production (Kulkarni et al., 2014). In contrast, analyses of the fatty acid composition of OMVs from *P. aeruginosa* revealed that these OMVs were enriched in longer and more saturated fatty acids compared with the outer membrane, suggesting that the more rigid regions of the outer membrane are prone to forming OMVs (Tashiro et al., 2011).

Bacterial membranes play a key role in mediating cellular and extracellular activities between living cells and their environment and in helping bacteria adapt to new conditions for their survival. It has been recently discovered that *C. ochracea* possesses unique properties of extracellular electron transfer (EET) to solid surfaces *via* their outer membrane (Zhang et al., 2020). OMVs are usually enriched with important OM characteristics. A recent study showed that OMVs released by the EET-capable environmental bacteria *Geobacter sulfurreduc*ens not only enhance EET but also confer electrogenic ability to non-EET capable strains and enhance the metabolism of other cells (*G. sulfurreducens* mutant strain ΔomcZ and *Escherichia coli*) (Liu et al., 2020). These functions are mainly attributed to the abundance of membrane-bound enzymes (cytochromes) bound to or entrapped in OMVs. Similarly, OMVs produced by *C. ochracea* may also mediate EET and influence the metabolism of other oral bacteria by electron transport over long distances. This ecologically important but overlooked biological (electron transfer) process requires further investigation.

Interactions between eukaryotic cells and vesicles from pathogenic bacteria suggest a role for vesicles in pathogenesis (Kuehn and Kesty, 2005). Therefore, OMVs released by oral pathogens not only contribute to the progression of periodontal disease but also develop systemic diseases. For example, *P. gingivalis* OMVs equipped with protease gingipains, that is, cargo proteins of vesicles, stimulate changes in glucose metabolism in the liver and contribute to the progression of diabetes mellitus (Seyama et al., 2020). In addition, a significant portion of *C. ochracea* development is harbored within oral polymicrobial biofilms. OMVs released from biofilms may be involved in a wide range of pathological processes to attack host cells. This OMV biogenesis study lacks data to explain the inclusive role of OMVs in pathogenesis. Therefore, further studies are needed to understand how *C. ochracea* OMVs manipulate the host immune response.

## Data Availability Statement

The original contributions presented in the study are included in the article/Supplementary Material, further inquiries can be directed to the corresponding author/s.

## Author Contributions

DN, WM, SS, ST, TS: conceptualization, methodology, data curation, writing – original draft preparation. NN: data discussion. MT: data analysis, reviewing and editing. AO: supervision, conceptualization, writing – reviewing and editing. All authors contributed to the article and approved the submitted version.

## Conflict of Interest

The authors declare that the research was conducted in the absence of any commercial or financial relationships that could be construed as a potential conflict of interest.
