# Supplementary material for: Biogenesis of Outer Membrane Vesicles Concentrates the Unsaturated Fatty Acid of Phosphatidylinositol in Capnocytophaga ochracea
Source: Front Microbiol. 2021 May 21;12:682685. doi: 10.3389/fmicb.2021.682685 (PMC8176214; doi:10.3389/fmicb.2021.682685)
Supplement: Supplementary file 1 [file Table_1.DOCX]

Supplementary Material


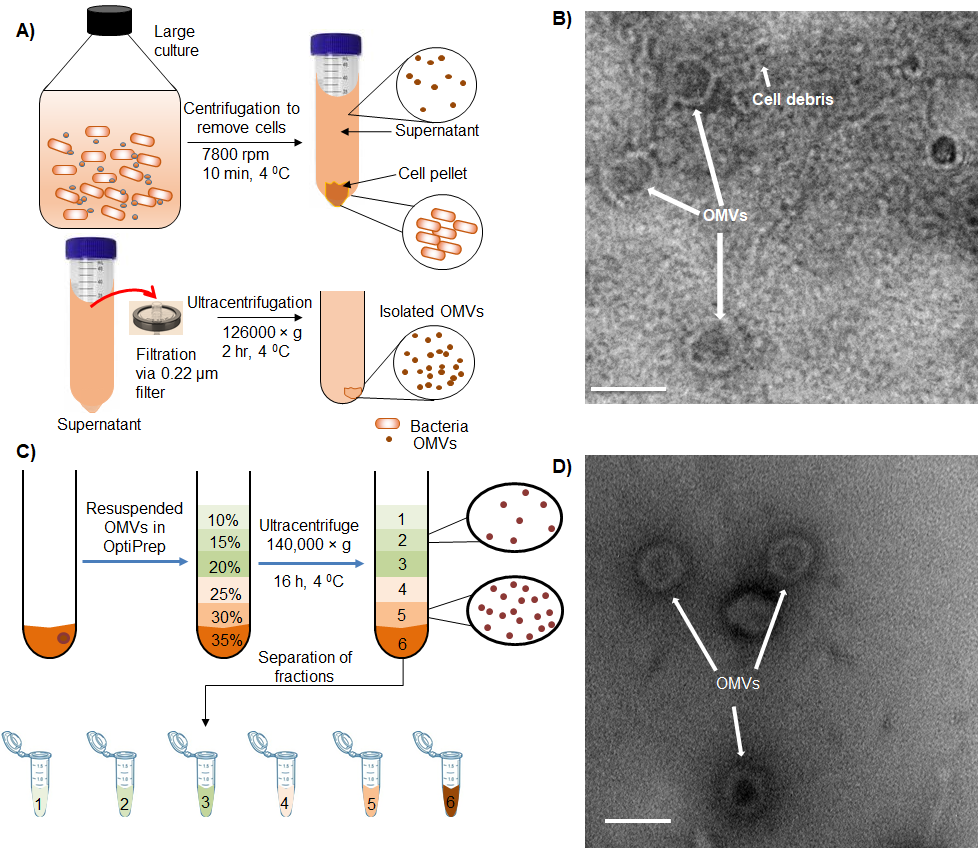


**Supplementary Figure S1. Isolation and TEM observations of OMVs. (A)** Protocol adopted for isolation of OMVs form *C. ochracea* cells. **(B)** Transmission electron microscopy image of OMVs stained with sodium molybdate. **(C)** Purification of OMVs by OptiPrep density gradient protocol. **(D)** TEM images of purified OMVs. Scale bar, 100 nm.

**
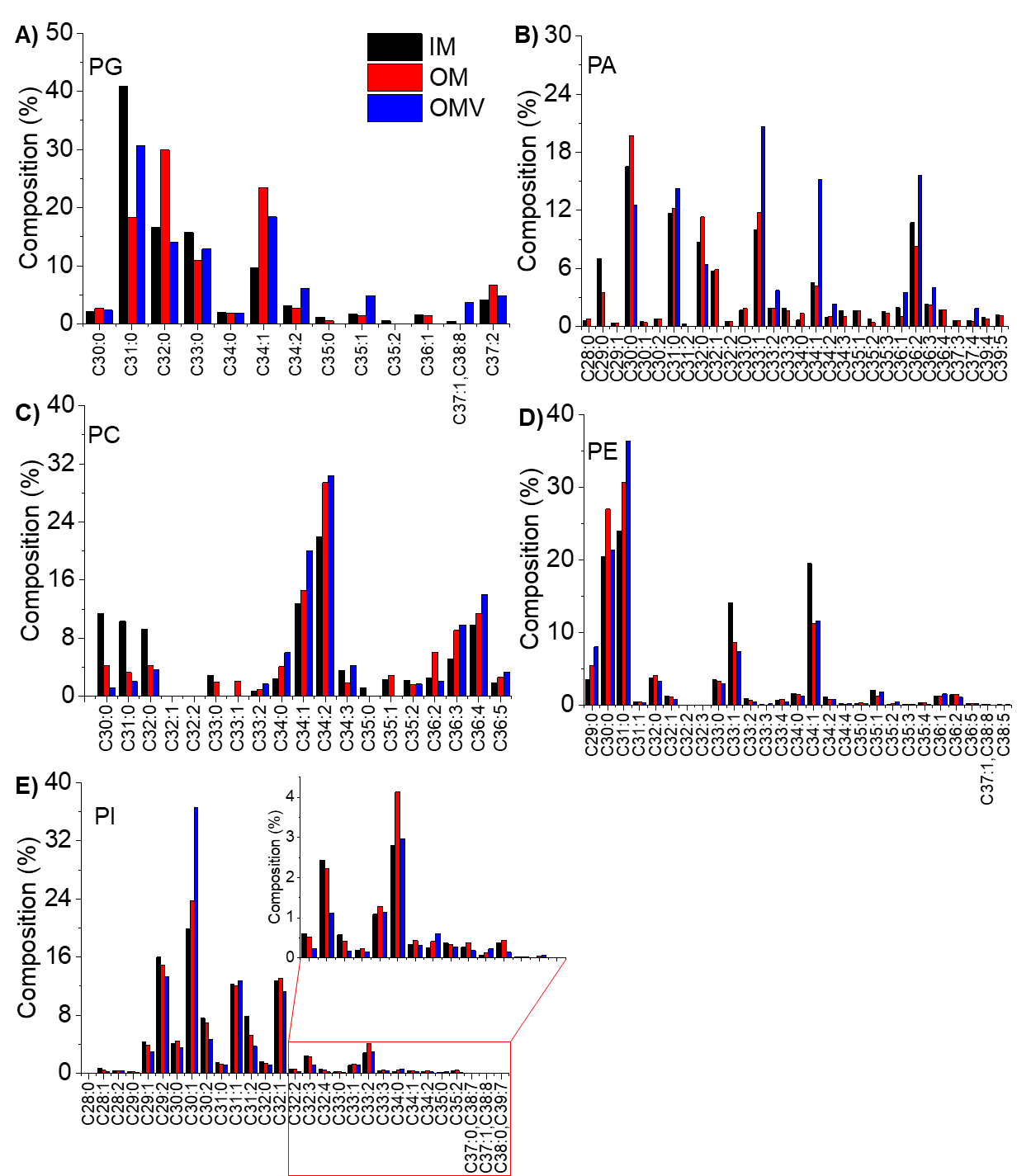
**

**Supplementary Figure S2. Fatty Acid Composition of Phospholipids in the IM, OM and OMVs of *P. gingivalis*** Percentage composition of fatty acids in phospholipids of **(A)** PG, **(B)** PA, **(C)** PC, **(D)** PE, and **(E)** PI. Inset of **(E)** is showing zoomed data of fatty acids from C32:2 to C38:0, C39:7. Fatty acids were identified by comparing their retention times with standards. Nonacosanoic acid (C29:0), Triacontanoic acid (C30:0), Hentriacontanoic acid (C31:0), Dotriacontanoic acid (C32:0), Tritriacontanoic acid (C33:0), Tetratriacontanoic acid (C34:0), Hexatriacontanoic acid (C36:0).

**Supplementary Table S1.** FM4-64 fluorescence intensity for each fraction of OMVs for different growth phases.

| Fraction number | pH 5.1 | | pH 5.3 | pH 5.7 |
| --- | --- | --- | --- | --- |
| 1 | | 0.509 | 0.624 | 0.43 |
| 2 | | 0.642 | 0.681 | 0.372 |
| 3 | | 0.489 | 0.662 | 0.743 |
| 4 | | 0.598 | 0.386 | 0.59 |
| 5 | | 0.685 | 0.285 | 0.648 |
| 6 | | 0.446 | 0.488 | 0.507 |
